# How Malpractice and Error Cases Influence Information Recall in General Practice Residents, a Vignette Study

**DOI:** 10.5334/pme.1730

**Published:** 2025-04-29

**Authors:** Charlotte van Sassen, Walter van den Broek, Patrick Bindels, Laura Zwaan

**Affiliations:** 1 Department of General Practice, Erasmus MC, University Medical Center Rotterdam, Rotterdam, The Netherlands; 2 Institute of Medical Education Research Rotterdam (iMERR), Erasmus MC, University Medical Center Rotterdam, Rotterdam, The Netherlands

## Abstract

**Purpose:**

Integrating diagnostic error and malpractice cases into clinical reasoning education may enhance diagnostic reasoning by highlighting atypical presentations and diagnostic risks in complex contexts. While emotionally engaging, these cases might also affect information retention. This study examines how malpractice, error, and neutral case presentations influence recall for different information types and their interaction with learners’ interest, satisfaction and anxiety levels.

**Methods:**

In this two-phase between-subjects experiment, 89 first-year general practice (GP) residents reviewed four clinical vignettes in either malpractice, diagnostic error, or neutral formats. Vignettes were structurally identical, with claim-related details in malpractice versions replaced by general medical information in others. Anxiety was measured pre- and post-exercise. After a one-hour filler task, participants completed a free recall task, and their interest and satisfaction levels were assessed. Recalled idea units (clinical case-specific, medical-theoretical, claim-specific) were analyzed using ANOVAs.

**Results:**

Anxiety, interest, and satisfaction levels remained similar across conditions. The proportion of total recalled idea units did not differ significantly (malpractice 11.38%, neutral 12.91%, error 13.12% *p* = 0.57). However, malpractice participants recalled fewer clinical case-specific units (malpractice 12.19%, neutral 19.43%, error 15.87% *p* = 0.007) while recalling more claim-specific units compared to medical-theoretical units in the other conditions (malpractice 7.23%, neutral 0.42%, error 1.3% *p* < 0.001).

**Conclusion:**

GP residents retained fewer clinical case-specific details from malpractice claim vignettes than from neutral vignettes, with the missing information substituted by claim-specific details, without an increase in anxiety or interest. Further research is needed to understand the long-term impact of these differences on future diagnostic accuracy in clinical practice.

## Introduction

Diagnostic errors are prevalent, with estimates of at least 5% of patients experiencing a diagnostic error in outpatient care in the US each year, which equals about 12 million adults annually [1]. Incorporating diagnostic errors and malpractice claims into medical education can enrich illness scripts and enhance diagnostic reasoning. While errors also occur in typical cases, our analysis of a claims database shows they often involve atypical presentations, rare diseases, or complex contextual factors [2]. Integrating these cases broadens exposure to diverse diagnostic challenges, enhancing the ability to recognize and manage both common and uncommon clinical scenarios [3,4,5,6]. Moreover, errors and malpractice claims may contribute to raising physicians’ awareness of the risk of diagnostic errors [2].

Malpractice cases often contain highly emotional content, which can activate readers’ emotional interest. Topics related to the human condition—such as power, death, money, injury, and sex—are universally compelling to readers [7] and residents often report that malpractice claims leave a profound impression on them [8]. High levels of interest and satisfaction with the learning material have been shown to enhance learning and memory [9,10,11,12,13,14]. However, research also indicates that adding highly interesting but unrelated content, known as *seductive details*, to scientific texts can lead to poorer memory retention for important content [15,16] while improving memory for the seductive details themselves [7,17,18,19]. This occurs because seductive details attract more visual attention, diverting focus from critical scientific concepts [15,16].

The effects of seductive texts may be influenced not only by interest-driven reading processes [10,11,19,20,21], but also by the emotional responses elicited by such content [7,22]. Reading about malpractice claims can trigger negative emotions like anger, guilt, insecurity, and fear [23,24,25]. The influence of intense emotions on learning has been extensively researched in educational and cognitive psychology [26,27,28]. While positive emotions generally enhance learning outcomes, negative emotions often have detrimental effects [29,30,31,32,33,34]. However, several studies demonstrate that negative emotions can also improve learning and retention by fostering more systematic processing of information, leading to detailed and item-specific analysis, whereas positive emotions are associated with heuristic processing, resulting in global and relational analysis [35,36,37].

While our previous studies found no direct differences in anxiety levels or future diagnostic performance of General Practice (GP) residents when exposed to erroneous or malpractice claim case vignettes [38,39], the effects of emotionally charged malpractice claim cases on information retention remain unclear. Understanding how information is processed could help determine whether, despite the absence of direct effects on anxiety and diagnostic performance, case presentation influences cognitive processes that may indirectly impact clinical reasoning and, ultimately, diagnostic performance in clinical practice. We hypothesize that malpractice claims generate greater interest and satisfaction with the learning material, potentially enhancing learning outcomes and performance. However, they may also increase anxiety, causing residents to retain more negative claim-specific information while recalling fewer relevant clinical case-specific details. This study examines whether differences exist in the amount and type of information (clinical case-specific, medical theoretic, claim-specific) remembered from cases presented as malpractice, neutral, or erroneous, and how these differences interact with anxiety, interest, and learners’ satisfaction in first-year GP residents. The findings will help assess whether there is a rationale for further research into the indirect effects of framing cases as neutral, error, or malpractice on diagnostic decision-making in clinical practice.

## Methods

All methods were carried out in accordance with relevant guidelines and regulations. Ethical approval was waived by the Medical Ethics Committee of Erasmus Medical Center (EMC) Rotterdam.

### Participants

Participants were first-year residents of GP vocational training at Erasmus Medical Center (EMC) in Rotterdam, The Netherlands.

### Setting

EMC is the largest of the eight academic hospitals in the Netherlands, employing nearly 18,000 staff members and handling approximately 670,500 outpatient consultations annually [40]. The Department of General Practice trains around 350 GP residents—14.7% of all GP trainees in the country—within a three-year curriculum, making it the largest GP training institution in the Netherlands [41]. Residents spend their first and third years in GP practices, while the second year includes placements in diverse settings such as hospital emergency departments, elder care institutions, mental health facilities, or specialized hospital departments. They work four days a week under supervision and participate in weekly teaching sessions, while their supervisors attend monthly sessions.

The GP training program integrates clinical reasoning education into both the scientific and practical aspects of its three-year curriculum, covering eight thematic areas throughout the training period. Weekly sessions are supervised by teaching staff, and daily clinical practice is conducted under the guidance of senior GPs. Clinical reasoning sessions use hypothetical case vignettes that address various diagnoses, primarily focusing on typical and common disease presentations in general practice.

### Study design

**Figure 1:**
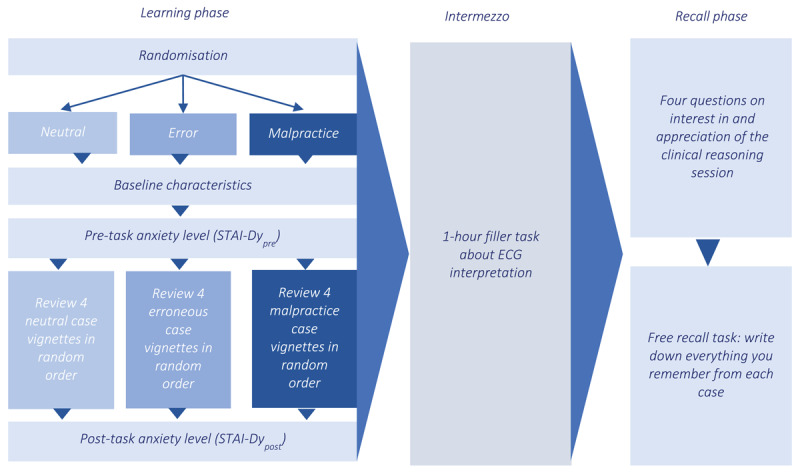
Study Design Overview for 89 First-Year GP Residents Participating in a Free Recall Task for 4 Neutral, Erroneous or Malpractice Clinical Case Vignettes, May 2021.

This two-phase, between-subjects experiment involved a learning phase and a recall phase, separated by a one-hour educational session on electrocardiograms as a filler task. Participants were randomly assigned to one of three conditions: neutral cases (N), cases with diagnostic errors (E), or cases with diagnostic errors and malpractice claims (M). Baseline characteristics and anxiety levels were measured before participants were tasked with reviewing four clinical vignettes in their assigned versions. Anxiety levels were measured again post-reviewing, followed by the filler task. Finally, in the recall phase, participants rated the clinical reasoning session and completed a free recall task for each case (see Figure 1).

### Materials and procedure

#### Claims database and case development

The primary liability insurer for GPs in the Netherlands provided anonymized data on diagnostic errors (malpractice claims) from 2012 to 2017 for educational and research purposes, without influencing the research process. Principal investigators selected four cases (tendon rupture, arterial occlusion, ablatio retinae, cerebrovascular accident) from the claims database, aligning with the educational priorities outlined in a prior study [2]. Corresponding anonymized files were obtained from the insurer and summarised into clinical vignettes, each in three versions: neutral, erroneous, and malpractice. In the neutral version, the case was presented without any additional commentary and the scenario unfolded correctly. The error version unfolded with an erroneous decision and the impact on the patient in the epilogue. The cause of this error was a cognitive error, as these are more relevant to the clinical reasoning process than, for example, system-related errors. The malpractice version added details about the resulting malpractice claim and financial compensation in the epilogue. To mitigate potential learning biases associated with vignette length, extraneous details in the erroneous and malpractice versions were replaced with general medical information about the disease in neutral cases and partly in the error cases (unrelated to the patient or the case). This ensured uniform word counts across all versions.

#### State-Trait Anxiety Inventory for measuring anxiety levels

Participants’ anxiety levels before and after solving the cases were measured using van der Ploeg’s Dutch version of the State-Trait Anxiety Inventory (STAI-DY or ZBV) [42,43,44]. This study used only the state anxiety section, consisting of 20 items rated on a 4-point Likert scale. The STAI state anxiety scale is highly responsive to immediate changes, capturing an individual’s current anxiety based on their responses to real-time contexts, like interventions or acute stressors [45]. Total scores range from 20 to 80, with higher scores indicating higher anxiety. Mean scores for Dutch students are 34.3 (males) and 35.2 (females) based on the original report [44]. Anxiety levels are classified as “no or low” (20–37), “moderate” (38–44), and “high” (45–80). The Dutch STAI-DY is reliable and valid, with a Cronbach’s alpha of 0.90 to 0.91 for state anxiety in Dutch students [44].

#### Procedure

This between-subjects clinical reasoning experiment was conducted in May 2021 during trainees’ regular plenary scientific educational session, which took place online due to COVID-19. Participants received a link to an online Qualtrics questionnaire for data collection and were unaware of the research aim during the sessions in order to prevent bias.

#### First session: Learning phase: Baseline data and pre- and post-case vignette exposure anxiety levels

In the learning phase, participants first provided baseline data (age, gender, GP residency start date, prior specialty experience, and duration in general practice). This information could be provided through free text or multiple-choice responses (see **Supplemental Digital Content Appendix 1**). Pre-task anxiety was measured using the STAI-DY. Participants then reviewed four clinical vignettes based on their assigned condition (neutral, error, or malpractice), with vignette order randomized (see **Supplemental Digital Content Appendix 2** for an example of a case vignette in three versions). Each participant received all four clinical vignettes presented in the same assigned condition (e.g., all four cases were neutral, erroneous, or included malpractice claims). The residents were asked to answer questions to induce deep processing of the information. The vignettes followed a serial cue method: participants received initial case information and responded to various questions, such as those pertaining to physical examination points of interest and management or diagnostic tests. As the case progressed, participants received additional information, asking them to formulate a likely diagnosis and differential diagnoses while revealing the case version’s specifics for each version (neutral unfolded correctly; erroneous unfolded with a diagnostic error and their impact; and malpractice added details about the resulting malpractice claim). The outcome of the case, including the correct diagnosis, was described in an epilogue. Responses to the questions were not analyzed, as the goal was to facilitate learning and reviewing the cases. Post-task anxiety was reassessed using the same method.

#### Second Session: Recall phase: Measuring learners’ interest and satisfaction and free recall task

In the second session, following the intermezzo with the ECG filler task, participants answered four general questions assessing their interest and satisfaction with the preceding clinical reasoning session. Subsequently, they were asked to write down in keywords everything they could remember from the four cases they reviewed in the learning phase, regardless of whether it was medical information, patient-related information or context factors (see **Supplemental Digital Content Appendix 3**).

#### Analysis

All calculations were done using SPSS Statistics version 25 for Windows (IBM). Differences were considered significant at *p* < 0.05 level.

#### First session: Learning phase: Baseline data and pre- and post-case vignette exposure anxiety levels

##### Baseline data and previous experience

Mean age (SD), gender distribution (%), time spent in GP residency and previous working experience yes/no (%) were calculated. The absolute and relative number of residents with previous working experience in a different specialization was calculated with the mean number of months [range] (SD) working in that field.

##### Anxiety levels (STAI-DY)

Total scores were calculated per participant. A mixed ANOVA with Bonferroni adjustment was conducted with moment of measuring (pre- vs post-reviewing the case vignettes) as within-subjects factor and vignette versions (neutral, error, malpractice) as between-subjects factor, to examine the effect of vignette version and moment of measuring on anxiety levels.

#### Second Session: Recall phase: Measuring learners’ interest and satisfaction and free recall task

##### Learners’ interest and satisfaction

The ratings for interest and satisfaction with the learning material were compared across the three conditions using a one-way ANOVA with post-hoc Bonferroni correction.

##### Free recall task

Two senior GPs independently categorized the idea units in the given case vignettes into clinical case-specific (all versions), claim-specific (malpractice version), and medical theory (neutral and error versions) (see Table 1). This categorization was based on the different texts used for each version of the case vignettes, each providing distinct information. For example, personal patient information (such as profession, age, social status, hobbies), main presenting complaints, the doctor’s management or treatment plan, and information on the course of the case were labeled as clinical case-specific. In contrast, information about the malpractice claim, such as the content of the complaint, the verdict of the claim, and the indemnity paid, was categorized as claim-specific information. Theoretical medical background information, such as prevalence rates, typical disease symptoms, or the general course and treatment of a disease, was labeled as medical theory. This last category only appeared in the neutral and partly in the error condition, as it was used to supplement the word count occupied by the description of the malpractice claim. There were few discrepancies, which were resolved through discussion.

**Table 1:** Word Count and Frequency of Types of Idea Units provided in Clinical Case Vignettes for 89 First-Year GP Residents Participating in a Free Recall Task for 4 Neutral, Erroneous or Malpractice Clinical Case Vignettes, May 2021.


CONDITION	CASE VIGNETTE	WORD COUNT	# IDEA UNITS		

			CASE-SPECIFIC	ADDITIONAL INFORMATION	

				CLAIM-SPECIFIC	MEDICAL THEORY

**Neutral**					

	Tendon Rupture	531	44	–	24

	Arterial Occlusion	614	72	–	45

	Ablatio Retinae	536	51	–	17

	Cerebrovascular Accident	666	63	–	34

**Error**					

	Tendon Rupture	537	55	–	22

	Arterial Occlusion	623	91	–	28

	Ablatio Retinae	537	60	–	8

	Cerebrovascular Accident	660	78	–	8

**Malpractice**					

	Tendon Rupture	539	51	20	–

	Arterial Occlusion	629	91	11	–

	Ablatio Retinae	529	60	10	–

	Cerebrovascular Accident	664	78	11	–


Based on the template categorization of the idea units in the given case vignettes, the idea units answered by the participants on the free recall task were subsequently categorized by a research assistant according to these types of idea units. Moreover, their accuracy was scored as “correct”, “incorrect”, or as “own interpretation,” where participants made conclusions or inferences based on the provided data that were not explicitly stated in the case but were logical and plausible within the context.

In order to relate the recalled idea units of the participants to the idea units provided in the case vignettes, mean proportions between the accuracy-scored recalled idea units and the provided idea units in the case vignettes were calculated for the total idea units, clinical case-specific idea units, and additional information idea units. These proportions were then compared across study conditions (neutral, erroneous, and malpractice) using a one-way ANOVA with post-hoc Bonferroni.

## Results

### Participants

The Qualtrics questionnaire link was sent to 114 participants, representing the full group that regularly attends the plenary educational sessions. Routine absences occur at these weekly sessions, and while privacy regulations prevent us from knowing specific reasons, the rates were typical. Since the study was part of a standard session, absences were likely due to factors like vacation, illness or parental leave, rather than the study itself, as participants were not informed in advance. Of the 109 participants that attended the session, ninety-three participants (85%) completed the first questionnaire and eigthy-nine participants (82%) completed both questionnaires of the learning- and recall phase. The participants were randomised into one of the three conditions: error (*N* = 28), malpractice (*N* = 29) or neutral (*N* = 32) (see **Supplemental Digital Content Appendix 4** for in- and exclusion information).

#### First session: Learning phase: Baseline data and pre- and post-case vignette exposure anxiety levels

##### Baseline data and previous experience

Baseline data on age, gender and previous experience collected in the first session are provided in the **Supplemental Digital Content Appendix 5** for each condition group. The participants were aged between 25 and 39 years (mean 29.74 years (2.70)) consisting of 27 males (30.3%) and 62 females (69.7%). Forty-two (47.7%) of the participants started their GP training within the last 3 months; forty-four (49.4%), within the last 9 months; three had a deviated starting time of 6, 15 and 129 months (mean 7.55 months (13.39)). Eighty-three (93.3%) participants had clinical experience before they started their GP residency with the majority in emergency medicine (39.3%), internal medicine (30.3%), and geriatrics (31.5%).

##### Pre- and post-case vignette exposure anxiety levels (STAI-DY)

**Figure 2:**
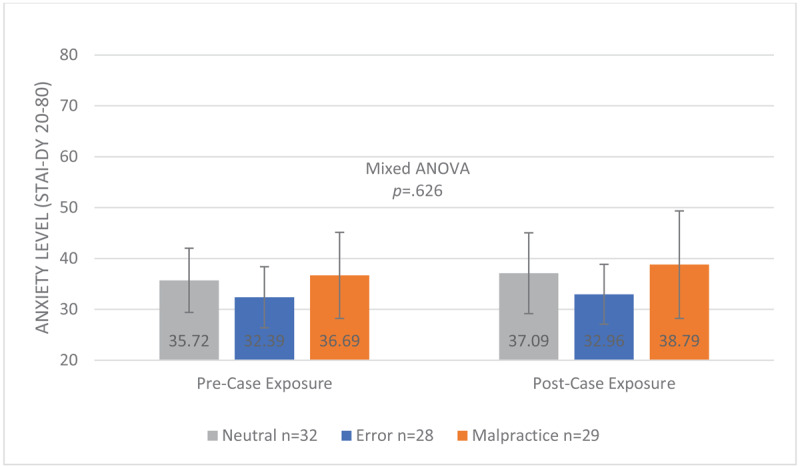
Mean Anxiety Levels (SD) Measured Pre- and Post-Exposure to Clinical Case Vignettes for 89 First-Year GP Residents Participating in a Free Recall Task for 4 Neutral, Erroneous or Malpractice Clinical Case Vignettes, May 2021.^a^ ^a^Measured with STAI-DY on a scale from 20–80, where a higher score indicates higher anxiety levels.

The mean STAI-DY levels before and after the exposure to the case vignette (reviewing the four vignettes in the first session) per study condition are provided in Figure 2. All fall into the “no or low anxiety” category [20,21,22,23,24,25,26,27,28,29,30,31,32,33,34,35,36,37], except for STAI_post_ of the malpractice group, which falls into “moderate anxiety” [38,39,40,41,42,43,44]. The mixed ANOVA showed no interaction effect for moment of measuring and case version *F*(2.86) = 0.470 *p* = 0.626 on anxiety levels, indicating that the effect of reviewing the case vignettes on anxiety levels was similar for the malpractice, error and neutral groups. However, a significant main effect of case version (*F*(2.86) = 3.874 *p* = 0.024) on anxiety levels was observed. This means that if timing of the measurement of anxiety levels (pre-/post-exposure to the case vignette, i.e. reviewing the case vignettes) is ignored, case version significantly influenced anxiety levels. The pairwise comparisons for the main effect of case version corrected using Bonferroni adjustments shows that the significant main effect reflects a significant mean difference of 5.063 between malpractice and error case versions with a medium effect size (SE 1.885 *p* = 0.026 95% CI [0.459–9.666], Cohen’s d 0.682) [46]. Moreover, there was a significant main effect of moment of scoring (*F*(1.86) = 4.548 *p* = 0.036 h^2^ = 0.050). This means that if all other variables are ignored, moment of scoring, i.e. solving the case vignettes, statistically significantly influenced anxiety levels with a small effect size (mean difference pre- post-exposure 1.350; SE 0.633, *p* = 0.036 95% CI [0.092–2.608], Cohen’s d 0.229) [46].

#### Second Session: Recall phase: Measuring learners’ interest and satisfaction and free recall task

##### Learners’ interest and satisfaction

**Figure 3:**
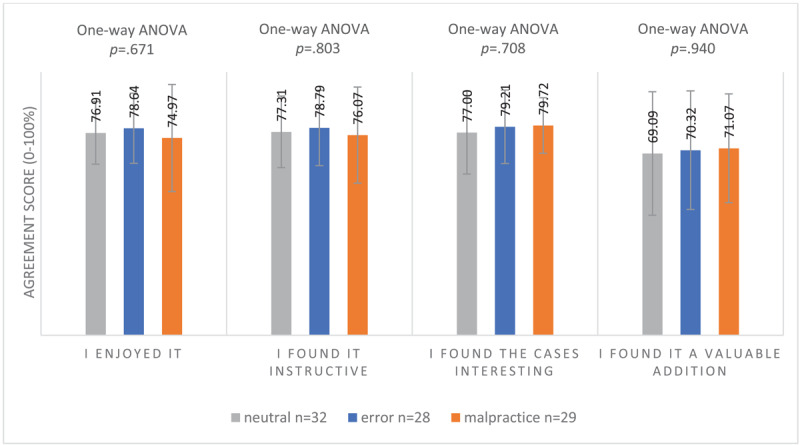
Mean Interest and Satisfaction Scores (SD) of Clinical Reasoning Session of 89 First-Year GP Residents Participating in a Free Recall Task for 4 Neutral, Erroneous or Malpractice Clinical Case Vignettes, May 2021.^a^ ^a^Agreement on a scale from 0–100% where 0% = not at all and 100% = very much.

GP residents showed no significant differences in interest or satisfaction across the different conditions (see Figure 3).

##### Free recall task

The majority of recalled idea units were remembered correctly, while the percentages of incorrectly and own interpretation idea units recalled were low (~<1%) and therefore practically irrelevant.

#### Total idea units (clinical case-specific + additional information)

The proportions of both correctly and incorrectly recalled total idea units did not differ significantly between conditions (total correct idea units: neutral 12.91%, error 13.12%, malpractice 11.38% (F(2.86) = 0.571, *p* = 0.567); total incorrect idea units: neutral 0.71%, error 0.66%, malpractice 0.84% (F(2.86) = 0.542, *p* = 0.584). The proportion of total own interpretation idea units recalled did differ significantly between conditions (F(2.86) = 4.495, *p* = 0.014). Specifically, the error condition had a higher proportion of own interpretation idea units (0.99%) compared to the neutral condition (0.43%; *p* = 0.013), but not compared to the malpractice condition (0.59%), as shown by post-hoc Bonferroni tests (see Figure 4a). However, these values were so low that they were deemed practically insignificant.

**Figure 4a:**
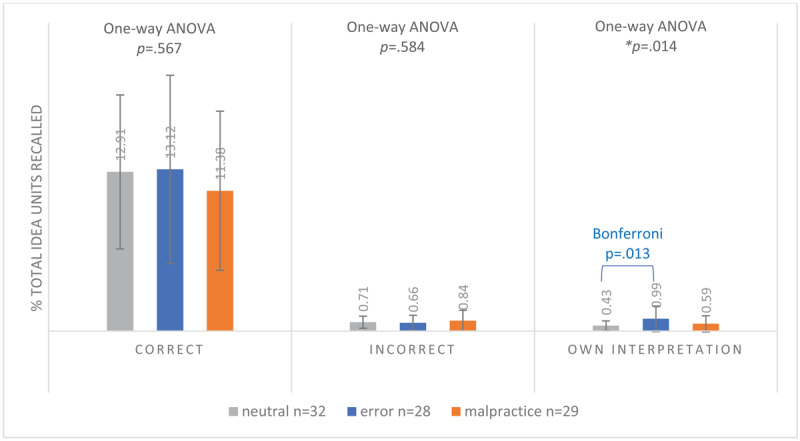
Mean Proportion of Recalled Correct, Incorrect and Own Interpretation Total Idea Units^a^ (SD) of 89 First-Year GP Residents Participating in a Free Recall Task for 4 Neutral, Erroneous or Malpractice Clinical Case Vignettes, May 2021. ^a^Clinical Case-Specific Idea Units Plus Additional Information Idea Units.

#### Clinical case-specific idea units

The proportion of correctly recalled clinical case-specific idea units differed significantly between the conditions (F(2.86) = 5.231, *p* = 0.007). Post-hoc Bonferroni tests revealed that the proportion was significantly higher in the neutral condition (19.43%) compared to the malpractice condition (12.19%, *p* = 0.005), but not compared to the error condition (15.87%). There was no significant difference between conditions for incorrectly recalled clinical case-specific idea units (F(2.86) = 0.885, *p* = 0.416). For own interpretation clinical case-specific idea units, numbers were again very low, but there was a significant difference between conditions (F(2.86) = 4.584, *p* = 0.013). The error condition recalled the highest proportion of these units (1.22%), which was significantly higher than both the neutral (0.64%, *p* = 0.039) and malpractice (0.58%, *p* = 0.023) conditions, as indicated by Bonferroni adjustments (see Figure 4b).

**Figure 4b:**
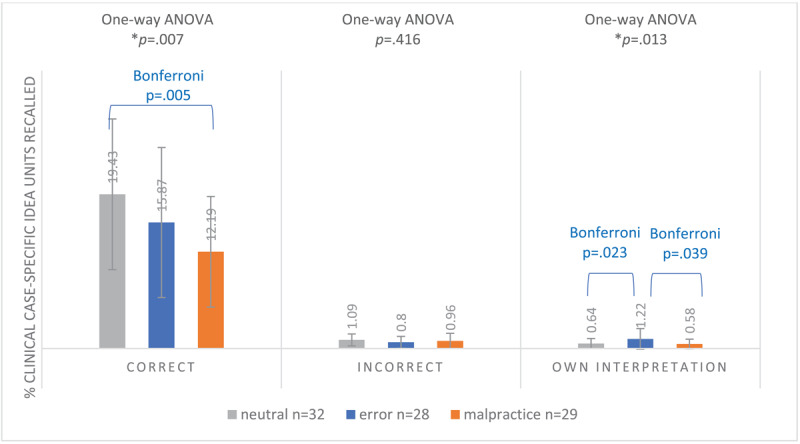
Mean Proportion of Recalled Correct, Incorrect and Own Interpretation Clinical Case-Specific Idea Units (SD) of 89 First-Year GP Residents Participating in a Free Recall Task for 4 Neutral, Erroneous or Malpractice Clinical Case Vignettes, May 2021.

#### Additional information idea units (claim-specific for malpractice; medical theory for error and neutral)

The proportion of correctly recalled additional information idea units differed significantly between conditions (F(2.86) = 22.352 *p* < 0.001). Post-hoc Bonferroni tests revealed that participants in the malpractice condition remembered significantly more correct claim-specific idea units (7.23%) compared to the correct medical theoretical idea units recalled in the error (1.3%, *p* < 0.001) and neutral (0.42%, *p* < 0.001) conditions. There was no significant difference in incorrect recalled idea units between conditions (F(2.86) = 2.239 *p* = 0.113). The proportions of own interpretation idea units also differed significantly between conditions (F(2.86) = 3.589 *p* = 0.032). The malpractice condition had a higher proportion of own interpretation idea units (0.66%) compared to the neutral (0.03%) and error (0.00%) conditions, although these numbers were very low (see Figure 4c).

**Figure 4c:**
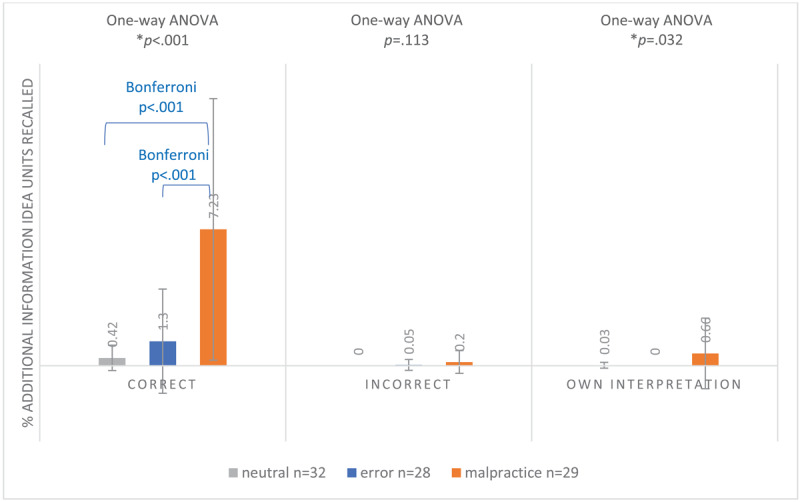
Mean Proportion of Recalled Correct, Incorrect and Own Interpretation Additional Information Idea Units^a^ (±SD) of 89 First-Year GP Residents Participating in a Free Recall Task for 4 Neutral, Erroneous or Malpractice Clinical Case Vignettes, May 2021. ^a^Claim-Specific Idea Units for malpractice clinical case vignettes; Medical Theoretical Idea Units for erroneous and neutral clinical case vignettes.

In summary, the proportion of total correct recalled idea units (clinical case-specific plus claim- or medical theory-specific) did not differ between conditions. However, in the malpractice condition, fewer correct clinical case-specific idea units were recalled compared to the neutral condition, with this gap being filled by claim-specific idea units.

## Discussion

This study demonstrates that GP residents exposed to clinical reasoning cases involving a malpractice claim recall less clinical case-specific information than those reviewing neutral cases. Instead, they retain more malpractice claim-specific details. The overall amount of information recalled was consistent across all case versions, with variations only in the type of information. While the absolute difference in the type of information recalled might appear small – the malpractice group remembered 7.24% less correct clinical case-specific information than the neutral group – this amounts to roughly a third (37.6%) less clinical information, which is substantial. This absolute reduction was almost balanced by an increase in correct claim-specific information recalled in the malpractice group, which was 6.81% higher compared to the correct medical-theoretical information retained by the neutral group, who only remembered 0.42% correct medical theoretical learning units. The proportions of recalled ‘incorrect’ and ‘own interpretation’ learning units were all very low (<1%), rendering the observed differences between the conditions practically irrelevant.

These findings support our hypothesis and align with literature suggesting that seductive details capture attention and result in poorer memory retention for essential content [15,16], while enhancing memory for the seductive details themselves [7,17,18,19]. The interaction between attention to seductive details during reading and deficits in learning can be partially explained by Cognitive Load Theory (CLT). CLT posits that the human cognitive system has limited working memory capacity. When working memory is overloaded with irrelevant but highly emotionally engaging information, such as seductive details, cognitive load increases, impeding learning and retention [47,48,49]. This theory has also been validated in medical education, where increased cognitive load has been shown to decrease trainees’ diagnostic performance in simulation trainings [32,33,50].

However, our study found no differences in evoked emotions or interest and satisfaction between conditions that could explain the variations in the types of information recalled from the case vignettes. While average anxiety levels increased by 1.35 after reviewing the vignettes—indicating a slight rise in stress that is likely clinically irrelevant on a scale from 20 to 80—this increase was consistent across all conditions, with no significant differences observed between them. Furthermore, the residents did not perceive the malpractice cases as more interesting or valuable. These findings align with our previous studies [38,39]. As a result, the mechanisms behind the differences in information retention across the case versions remain unclear.

However, a more critical question arises from our conclusions: what are the implications of remembering different types of information from malpractice claims? Does it affect future diagnostic accuracy in clinical practice? Our previous research has indicated that future diagnostic accuracy does not appear to be affected by malpractice claims [38,39]. Thus, despite recalling fewer details related to disease presentation, symptoms, or vital information needed for diagnosis, residents do not seem to exhibit decreased future diagnostic accuracy when malpractice cases are used as clinical reasoning case vignettes. This prompts the question: are the seductive details about malpractice claims in a clinical vignette truly “irrelevant” for clinical reasoning? It is possible, for example, that residents mentally link claim details with essential diagnostic information, thereby expanding the disease-specific illness scripts [51] and increasing alertness and awareness of diagnostic errors related to these diagnoses. These expanded illness scripts may not only encompass typical clinical features for pattern recognition, but also contextual risks, atypical presentations and additional information associated with errors or claims, fostering increased vigilance regarding diagnostic errors in future cases in clinical practice. This heightened vigilance could encourage reflection or cognitive cross-referencing, where residents actively question whether they are overlooking important details that could lead to a misdiagnosis [52,53]. This could result in more thorough diagnostic evaluations, potentially reducing the likelihood of errors without necessarily requiring recall of every clinical case-specific detail. Moreover, this type of context-driven alertness may enhance adaptive expertise [54,55], that is, of balancing efficiency and innovation in clinical problem solving [56], allowing clinicians to apply a flexible, nuanced approach in novel situations rather than relying solely on rote memory. These processes could ultimately help maintain or enhance future diagnostic accuracy in clinical practice, even when fewer specific case details are remembered. Furthermore, more memorable, impressive (negative) claim-specific information may be retained longer, potentially affecting long-term memory [57]. Future research should explore how varying recall patterns influence diagnostic decision-making and patient outcomes in clinical practice. Understanding this can help educators assess the potential value of incorporating erroneous and malpractice claims into clinical reasoning education, while also guiding them in balancing the types of cases presented to trainees.

### Limitations

Several limitations may explain the absence of differences in anxiety levels across case conditions. First, the use of paper-based cases may not fully capture the emotional intensity of real-life scenarios, which could elicit stronger emotional responses. Additionally, learning from others’ errors may affect participants differently than learning from their own. Moreover, first-year residents, who are not yet working independently and unsupervised, might not feel the full pressure of malpractice claims, resulting in less anxiety. It is also possible that the claim details did not evoke a strong enough emotional response to be measurable. However, the increased recall of claim-specific information—compared to the less engaging medical-technical theory—suggests that the cases were sufficiently engaging. Finally, the STAI-DY questionnaire may not have been the most suitable tool for measuring the specific anxiety relevant to this study or lacked the sensitivity to detect subtle differences over a short time span.

Another limitation is that we did not assess differences in recall across learner levels (e.g., beginner, intermediate, or advanced). As a result, our findings may not be generalizable to other learner populations, and we cannot determine how variations in experience or expertise might affect recall or clinical reasoning processes.

### Conclusion

This study demonstrates that GP residents recall about a third fewer clinical case-specific details from vignettes that include a malpractice claim description compared to neutral cases, which is substituted by malpractice claim-specific details, without heightening anxiety or enhancing satisfaction of the learning material. The mechanisms behind these differences in memory retention, as well as their long-term impact, such as effects on future diagnostic accuracy in clinical practice, remain unknown and require further research.

## Data Accessibility Statement

The datasets used and/or analyzed during the current study are available from the corresponding author on reasonable request.

## Additional Files

The additional files for this article can be found as follows:

10.5334/pme.1730.s1 Supplemental Digital Content. Appendices 1 to 5.

## Figures and Tables

**Table:** 

**CRE**	Clinical Reasoning Education
**CLT**	Cognitive Load Theory
**GP**	General Practitioner
**STAI-DY**	State-Trait Anxiety Inventory
**N**	Neutral
**E**	Error
**M**	Malpractice
**EMC**	Erasmus Medical Center
