# Supplementary material for: How Malpractice and Error Cases Influence Information Recall in General Practice Residents, a Vignette Study
Source: Perspect Med Educ. 2025 Apr 29;14(1):194–207. doi: 10.5334/pme.1730 (PMC12047635; doi:10.5334/pme.1730)
Supplement: Supplemental Digital Content. — Appendices 1 to 5. [file pme-14-1-1730-s1.pdf]

Supplemental Digital Content

Appendix 1

Questionnaire on baseline data and previous working experience for 89 First-Year GP Residents Participating in a Free Recall Task for 4 Neutral, Erroneous or Malpractice Clinical Case Vignettes, May 2021

-What is your age? YY

-What is your gender? M/F

-When did you start GP training? MM/YYYY

-Did you have previous clinical experience before you started GP training? Yes/ No

-If yes: Please select in which speciality you have previous clinical experience (multiple answers possible) and how many months:

Internal medicine

Emergency medicine

Pediatrics

Surgery

Gynaecology and obstetrics

Neurology

Cardiology

Dermatology/Ear nose throat/ Eye

Geriatrics

Psychiatry

Intensive care

Tropical medicine

Military services

Other <free text>

Appendix 2

Example of clinical case vignette in 3 versions for 89 First-Year GP Residents Participating in a Free Recall Task for 4 Neutral, Erroneous or Malpractice Clinical Case Vignettes, May 2021

Tendon Rupture, Neutral version 531 words

Mr. Berendse is a 44-year-old administrative assistant at an office. He presents to the general practitioner with acute pain in his right calf, which occurred after a misstep during a game of badminton. He describes making a hyperextension movement, hearing a snap, and experiencing immediate severe pain.

What do you look for during a physical examination?

The GP on duty examines Mr. Berendse. There is tenderness in the calf and Achilles tendon without hematoma formation. He can barely walk on it. There is no palpable gap (delle) in the tendon.

What do you suspect?

Initially, the GP considers a "coup de fouet" injury (calf strain), for which the treatment includes rest, cooling, ibuprofen, and possibly a heel lift. During the consultation, the patient mentions that he is scheduled to travel to the Philippines in 2.5 weeks and is concerned about whether he will be able to make the flight to Singapore, as he is currently barely able to walk. The GP still has some doubts about your initial diagnosis of a calf strain and decides to perform the Thompson test. This test is positive (i.e., no plantar flexion when squeezing the calf muscle).

What is your next step?

What is your most likely diagnosis?

If applicable, what are your differential diagnoses?

Epilogue: Based on the positive Thompson test, the GP immediately orders an ultrasound, suspecting an Achilles tendon rupture. The ultrasound reveals a thickened Achilles tendon on the right compared to the left, consistent with pre-existing tendinosis. The Achilles tendon is torn at its muscle attachment. The patient is promptly referred to an orthopedic surgeon. Due to the complete rupture, the tendon is surgically repaired, and Mr. Berendse's lower leg is placed in a cast in a pointed position (equinus position) for 6 weeks. He also receives thrombosis prophylaxis with heparin for 6 weeks. Unfortunately, this means that Mr. Berendse's trip to the Philippines cannot go ahead.

An Achilles tendon rupture typically occurs in men between the ages of thirty and forty, who are often moderately trained or unprepared for a match. There is often a classic story: a sudden, rapid muscle contraction causes severe pain at the lower back of the calf, and the patient often thinks they have been kicked in the calf or heel. Walking becomes nearly impossible, and there is no strength to stand on the toes. The diagnosis can be made through physical examination. A complete rupture is often less painful than a partial one. In a partial rupture, there is still tension on the tendon and the tear. In an Achilles tendon rupture, the patient cannot stand on their toes. On palpation, a gap can be felt at the site of the rupture, and sometimes the torn end of the tendon can be felt as a lump under the skin. The Thompson test is positive: squeezing the calf muscle does not result in plantar flexion. Treatment can be either conservative, with a cast/splint with the foot in a pointed position, or surgical, with primary suturing of the tendon followed by cast treatment. During the first 6 weeks after the injury, there is an indication for medication prophylaxis to reduce the risk of thrombosis in the leg or pulmonary embolism, using heparin.

Tendon Rupture, Error version, 537 words

Mr. Berendse is a 44-year-old administrative assistant. He visits the general practitioner with acute pain in his right calf, which occurred after a misstep during a game of badminton. He reports making a hyperextension movement, hearing a snap, and experiencing immediate severe pain.

What do you look for during a physical examination?

The GP on duty examines Mr. Berendse. There is tenderness in the calf and Achilles tendon without hematoma formation. He can barely walk.

What do you suspect?

Suspecting a calf strain ("coup de fouet"), the GP prescribes rest, cooling, ibuprofen, and possibly a heel lift.

Eleven days later, Mr. Berendse visits his regular GP due to persistent pain in his calf. He is concerned because he is scheduled to travel to the Philippines in 2.5 weeks, requiring a flight to Singapore. Over the past few days, he has managed to mobilize himself and move independently, though with great difficulty. During physical examination, there is swelling of the calf and some yellow discoloration of the foot. There is no palpable gap (delle) in the calf or Achilles tendon, but the entire calf is tender up to the knee, with the most tenderness just above the heel. The Thompson test is positive (i.e., no plantar flexion when squeezing the calf muscle).

What is your next step?

What is your most likely diagnosis?

If applicable, what are your differential diagnoses?

Epilogue: The GP orders an ultrasound, suspecting an Achilles tendon rupture. The ultrasound reveals a thickened Achilles tendon on the right compared to the left, consistent with pre-existing tendinosis. The Achilles tendon is torn at its muscle attachment. Additionally, a deep vein thrombosis (DVT) is detected in several calf veins.

For the thrombosis, he is treated according to the DVT protocol with Fragmin and acenocoumarol for 3 months. Due to the delay in diagnosis, primary surgical repair of the tendon is no longer possible. Instead, Mr. Berendse’s lower leg is placed in a cast in a pointed position (equinus position) for 6 weeks. Unfortunately, this means that Mr. Berendse’s trip to the Philippines cannot go ahead.

An Achilles tendon rupture typically occurs in men between the ages of thirty and forty, who are often moderately trained or unprepared for a match. The condition often follows a classic pattern: a sudden, rapid muscle contraction causes severe pain at the lower back of the calf, and the patient often thinks they have been kicked in the calf or heel. Walking becomes nearly impossible, and there is no strength to stand on the toes. The diagnosis can be made through physical examination. A complete rupture is often less painful than a partial one. In a partial rupture, there is still tension on the tendon and the tear. Palpation may reveal a gap at the site of the rupture, and sometimes the torn end of the tendon can be felt as a lump under the skin. The Thompson test is positive: squeezing the calf muscle does not result in plantar flexion. Treatment can be either conservative, with a cast/splint in the pointed position, or surgical, with primary suturing of the tendon followed by cast treatment. During the first 6 weeks after the injury, thrombosis prophylaxis with heparin is indicated.

Tendon Rupture, Malpractice version, 539 words

Mr. Berendse is a 44-year-old administrative assistant. He visits the general practitioner (GP) with acute pain in his right calf, which occurred after a misstep during a badminton game. He reports making a hyperextension movement, hearing a snap, and experiencing immediate severe pain.

What do you look for during a physical examination?

The GP on duty examines Mr. Berendse. There is tenderness in the calf and Achilles tendon without hematoma formation. He can barely walk.

What do you suspect?

Suspecting a calf strain ("coup de fouet"), the GP prescribes rest, cooling, ibuprofen, and possibly a heel lift.
Eleven days later, Mr. Berendse visits his regular GP due to persistent pain in his calf. He is concerned because he is scheduled to travel to the Philippines in 2.5 weeks, requiring a flight to Singapore. Over the past few days, he has managed to mobilize himself and move independently, though with difficulty. During physical examination, there is swelling of the calf and some yellow discoloration of the foot. There is no palpable gap (delle) in the calf or Achilles tendon. However, the entire calf is tender up to the knee, especially just above the heel.

What is your next step?
What is your most likely diagnosis?
If applicable, what are your differential diagnoses?

The Thompson test is positive (i.e., no plantar flexion when squeezing the calf muscle). The GP orders an ultrasound, suspecting an Achilles tendon rupture. The ultrasound reveals a thickened Achilles tendon on the right compared to the left, consistent with pre-existing

tendinosis. The Achilles tendon is torn at its muscle attachment. Additionally, a deep vein thrombosis (DVT) is detected in several calf veins.

For the thrombosis, he is treated according to the DVT protocol with Fragmin and acenocoumarol for 3 months. For the Achilles tendon rupture, Mr. Berendse’s lower leg is placed in a cast in a pointed position (equinus position) for 6 weeks.

Almost six months after the incident, the patient holds the attending GP at the emergency out-of-hours service responsible for missing the diagnosis of Achilles tendon rupture, which led to the development of calf vein thrombosis. The claim is based on negligent care, citing insufficient physical examination (e.g., Thompson’s test, plantar flexion, and resistance testing, as well as side-to-side comparison) and failure to properly instruct the patient to visit his regular GP if mobilizing or bearing weight was problematic. At least, this is not recorded in the patient's file. A timely diagnosis could have allowed for primary tendon repair and immediate initiation of thrombosis prophylaxis, reducing the risk of thrombosis. This liability is acknowledged, and a compensation of nearly €3000 is awarded. Additionally, the patient claims damages for a missed holiday due to being unable to travel to the Philippines three weeks after the incident. However, this part of the claim is not honored, as even with prompt surgical repair, he would have been in a lower leg cast, and circular casting is not recommended during a long flight due to cabin pressure differences. Given the necessary equinus position, there was no option for removable casting or splinting, even with primary repair. Considering the thrombosis and the short time frame for proper anticoagulation adjustment with acenocoumarol, flying would have been discouraged anyway.

Appendix 3

Questionnaire of Interest and Acceptance of Clinical Reasoning Session and Recall Task for 89 First-Year GP Residents Participating in a Free Recall Task for 4 Neutral, Erroneous or Malpractice Clinical Case Vignettes, May 2021

You participated in the clinical reasoning session on the conditions: tendon rupture, arterial occlusion, ablatio retinae and cerebrovascular accident.

Please answer a few statements about the clinical reasoning exercise in general. Please specify your answer on a scale of 0 to 100%, where 0% is 'I do not agree at all' and 100% is 'I agree very much'.

-I enjoyed doing this clinical reasoning exercise

-I found it instructive to do this clinical reasoning exercise

-I found the cases interesting

-I found it a valuable addition to the educational program

On the following pages, we ask you to write down in keywords EVERYTHING that you remember for each case. This does not have to be only medical information, but also, for example, patient-related information or context factors.

-*tendon rupture* <free text>

-*arterial occlusion* <free text>

-*ablatio retinae* <free text>

-*cerebrovascular accident* <free text>

Appendix 4

In- and exclusion of participants for 89 First-Year GP Residents Participating in a Free Recall Task for 4 Neutral, Erroneous or Malpractice Clinical Case Vignettes, May 2021

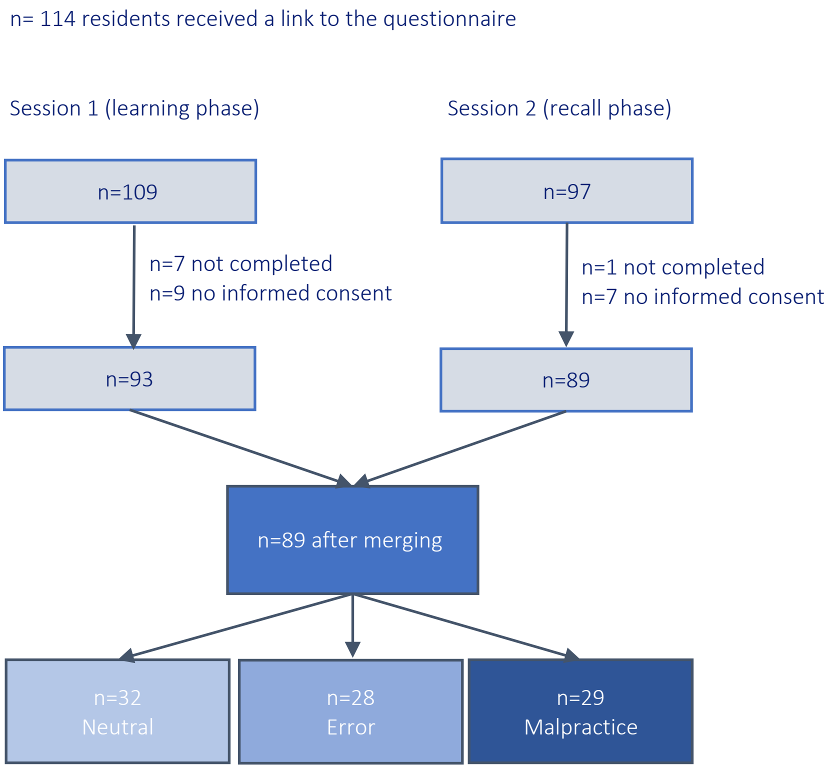

Appendix 5

Baseline Data of 89 First-Year GP Residents Participating in a Free Recall Task for 4 Neutral, Erroneous or Malpractice Clinical Case Vignettes, May 2021

	**Total n= 89**	**Neutral n=32**	**Error n= 28**	**Malpractice n=29**
**Mean age (SD)**	29.74 (2.70) years	39.66 (2.68) years	30.30 (2.95) years	29.31 (2.48) years
**Gender (%)**	62 (69.7%) female<br>27 (30.3%) male	23 (71.9%) female<br>9 (28.2%) male	17 (60.7%) female<br>11 (39.3%) male	22 (75.9%) female<br>7 (24.1%) male
**Previous experience (%)**	83 (93.3%) yes<br>6 (6.7%) no	31 (96.9%) yes<br>1 (3.1%) no	26 (92.9%) yes<br>2 (7.1%) no	26 (89.7%) yes<br>3 (10.3%) no
